# Variability in conditioned pain modulation predicts response to NSAID treatment in patients with knee osteoarthritis

**DOI:** 10.1186/s12891-016-1124-6

**Published:** 2016-07-13

**Authors:** Robert R. Edwards, Andrew J. Dolman, Marc. O. Martel, Patrick H. Finan, Asimina Lazaridou, Marise Cornelius, Ajay D. Wasan

**Affiliations:** Department of Anesthesiology, Harvard Medical School, Brigham & Women’s Hospital, 850 Boylston St, Suite 302, Chestnut Hill, MA 02467 USA; Department of Psychiatry, Johns Hopkins University School of Medicine, 5510 Nathan Shock Drive, Suite 100, Baltimore, MD 21224 USA; Departments of Anesthesiology and Psychiatry, University of Pittsburgh School of Medicine, 400 Centre Ave, #400, Pittsburgh, PA 15206 USA; Pain Management Center, Brigham & Women’s Hospital, 850 Boylston St, Chestnut Hill, MA 02467 USA

**Keywords:** Pain, Neuropathic, Osteoarthritis, NSAID, Diclofenac, Quantitative sensory testing, Conditioned pain modulation

## Abstract

**Background:**

Patients with painful knee osteoarthritis (OA) demonstrate hyperalgesia and altered pain-modulatory responses. While some prior work has demonstrated cross-sectional associations between laboratory and clinical pain measures, it is unknown whether individual variability in quantitative sensory testing (QST) responses at baseline can prospectively predict analgesic treatment responses.

**Method:**

Patients with knee OA (*n* = 35) were compared on QST responses to a demographically-matched pain-free control group (*n* = 39), after which patients completed a month-long treatment study of diclofenac sodium topical gel (1 %), applied up to 4 times daily.

**Results:**

OA patients demonstrated reduced pain thresholds at multiple anatomic sites, as well as reduced conditioned pain modulation (CPM) and enhanced temporal summation of pain. The most pain-sensitive patients tended to report the most intense and neuropathic OA pain. Following diclofenac treatment, the knee OA cohort showed a roughly 30 % improvement in pain, regardless of the presence or absence of neuropathic symptoms. Baseline CPM scores, an index of endogenous pain-inhibitory capacity, were prospectively associated with treatment-related changes in clinical pain. Specifically, participants with higher CPM at baseline (i.e., better functioning endogenous pain-inhibitory systems) showed more reduction in pain at the end of treatment (*p* < .05).

**Conclusions:**

These results support prior findings of amplified pain sensitivity and reduced pain-inhibition in OA patients. Moreover, the moderate to strong associations between laboratory-based measures of pain sensitivity and indices of clinical pain highlight the clinical relevance of QST in this sample. Finally, the prospective association between CPM and diclofenac response suggests that QST-based phenotyping may have utility in explaining inter-patient variability in long-term analgesic treatment outcomes.

**Trial registration:**

ClinicalTrials.Gov Identifier: NCT01383954. Registered June 22, 2011.

## Background

Knee osteoarthritis (OA) is a highly prevalent cause of persistent pain and disability throughout the world [1]. The pathophysiology of OA pain is complex, with contributions of peripheral factors such as synovial inflammation, as well as central and peripheral nervous system sensitization. Individuals reporting persistent OA-related pain are characterized by enhanced pain sensitivity on quantitative sensory testing (QST), and amplified brain responses to noxious stimuli [2, 3]. Similar to other chronic pain conditions, there is broad inter-patient variability in pain symptomatology, including neuropathic pain symptoms [4, 5]. Studies have found differing degrees of association between radiographic changes and pain severity, but it is generally accepted that factors other than radiographic features affect pain levels [2, 6, 7]. Of note, numerous pain researchers have theorized that differing degrees of sensitization of central nociceptive circuits may explain why some patients are disabled by severe OA pain and prominent neuropathic symptoms, while others with similar radiographic features report minimal pain and few functional difficulties [2, 3, 8].

QST refers to a set of psychophysical methods used to quantify somatosensory function. It is based on measuring responses to calibrated somatosensory stimuli and represents an extension and refinement of the bedside clinical examination [3, 8]. QST has been used for: diagnosing sensory neuropathies, investigating pain mechanisms, characterizing somatosensory profiles, and quantifying individual differences in pain sensitivity [9]. In addition to indices of pain sensitivity such as pain threshold, many QST protocols also evaluate pain-modulatory processes such as conditioned pain modulation (CPM) and temporal summation of pain. These dynamic measures of pain-inhibitory and pain-facilitatory processes can provide important information about the central nervous system’s processing and modulation of pain-related information [8, 9].

To date, a handful of cross-sectional QST studies in knee OA patients report evidence of amplified pain sensitivity at affected joint sites and at unaffected body locations [10]. Moreover, several longitudinal reports have indicated that disease-modifying treatments such as joint replacement can reverse the observed sensitization and “normalize” QST profiles [11, 12]. What is currently unknown, however, is how inter-patient variability in pain sensitivity and pain modulation might prospectively predict responses to analgesic treatments. This is an important area of ongoing work, but at present we are unaware of any relevant studies with knee OA patients, though there is increasing interest in this topic in OA [13, 14].

Diclofenac is an anti-inflammatory drug that inhibits prostaglandins; it is frequently used in the treatment of rheumatic disease [15, 16]. Non-steroidal anti-inflammatory drugs (NSAIDs), including diclofenac, are considered first line medication options for treating osteoarthritis pain. For knee OA, a common pain treatment is with oral or topical NSAIDS such as topical diclofenac gel (Voltaren Gel®) [15, 16]. A recent meta-analysis showed that topical diclofenac modestly but significantly improved pain and function scores over 12 weeks in patients with osteoarthritis of the knee [17]. We used QST to profile pain responses among patients with knee OA, and investigated the potential for pre-treatment QST responses to predict diclofenac’s treatment effectiveness. Our primary hypothesis was that diclofenac gel would improve knee pain, and that baseline variability in QST-assessed indices of hyperalgesia and pain modulation would predict the magnitude of treatment-related changes in knee pain, as has been observed for other treatments [18].

## Methods

### Design and participants

We first compared 35 knee OA patients to a demographically-comparable group of 39 pain-free controls using QST. This comparison was followed by a 5-week, uncontrolled effectiveness study of diclofenac gel in patients with knee OA (ClinicalTrials.Gov Identifier: NCT01383954). Logistically, the study consisted of 3 in-person visits: a screening visit followed by a one-week pain baseline assessment period using daily electronic diary entries, QST testing at a baseline visit following the week of electronic diary completion, and then a final end-of-treatment visit after 4 weeks of diclofenac gel use. The end-of-treatment visit included a QST session identical to that performed at baseline. Eligible patients were 30–75 years old and had radiographically-confirmed knee OA of Grade II or III. Potential participants were excluded for [1]: a history of meniscal or ACL involvement [2], current SNRI, tricyclic antidepressant, or anticonvulsant use [3], advanced renal disease [4], aspirin or NSAID hypersensitivity [5], congestive heart failure [6], a coagulation disorder [7], a history of ulcers or GI bleeding, and [8] pregnancy. Additionally, participants were required to stop any oral or topical NSAIDS for 1 week prior to enrollment.

Subjects were recruited by email advertising, web and bulletin board announcements in Boston, MA. Controls were demographically matched to the patient group, and were subject to the same exclusion criteria, with the additional criterion that controls were free from painful osteoarthritis or other chronic pain conditions.

The study medication was diclofenac sodium topical gel (1 %, Voltaren Gel®); consistent with the product labeling, subjects were instructed to apply the gel up to 4 times daily, using a total of no more than 32 g of diclofenac gel per day. Compliance with gel application was monitored using an electronic diary. Subjects were told to avoid bathing for 1 h after application and also to avoid the sun when using the gel.

In total, 137 volunteers (85 with OA, and 52 healthy adults) responded to advertisements. Of the initial 52 healthy adults, 39 expressed interest, were eligible, and were enrolled. Of the initial 85 OA patients, 61 expressed interest and were screened by phone. Out of these 61 interested candidates, 9 participants were ruled out at the screening visit for failing to meet eligibility criteria and thus, 52 subjects were finally enrolled in the study. The study was performed in accordance with the Declaration of Helsinki, and all study procedures were approved by the Brigham and Women’s Hospital Institutional Review Board. Written informed consent was obtained from every subject by study staff at the time of enrollment. Four subjects withdrew from the study, and 13 participants failed to provide adequate post-treatment pain rating data, and were thus excluded from the statistical analysis. Therefore, the final analytic sample included 35 OA patients.

### Measures

Patients completed multiple validated self-report measures of pain and function:

#### Knee Injury and Osteoarthritis Outcomes Score (KOOS)

This is a widely-used and well-validated self-report scale assessing activity-related knee OA pain [19]. Scores are normalized from 0–100, with higher scores indicative of less pain and better functioning.

#### Neuropathic pain

Participants rated the presence and intensity of 3 hallmark descriptors of neuropathic pain: burning pain, shooting pain, and sensitivity to touch [20, 21]. These items were rated from 0 = never to 100 = worst pain imaginable.

#### Electronic diaries

Patients monitored daily pain intensity, KOOS ratings of activity-related pain, and neuropathic symptoms, with Hewlett Packard © IPAQ electronic diaries [22]. Initial daily ratings were made before the first gel application; then, participants were prompted to rate their pain prior to as well as 30, 60, 120, and 240 min following their first gel application of the day. The initial daily rating and the pain ratings prior to each application were averaged within days and across days, providing weekly average pain intensity scores that reflected the mean pain intensity prior to gel applications.

### Quantitative sensory testing

For QST visits, participants refrained from intense physical exercise, using nicotine, or using the diclofenac gel for at least 4 h prior to testing. Clinical pain ratings (on a 0–100 scale) and verbal ratings of anxiety (on a 0–100 scale, with “no anxiety” and “severe anxiety” as the respective anchors) were obtained at several points during QST. During the roughly 45-min session, subjects were seated comfortably in a reclining chair.

Mechanical pain thresholds were assessed using a digital pressure algometer (Somedic; Sollentuna, Sweden). Pressure pain thresholds (PPThs) were determined twice, bilaterally at the trapezius muscle, the patella, and the metacarpophalangeal joint of the thumb. At each site, mechanical force was applied using a 0.5 cm^2^ probe covered with polypropylene pressure-transducing material; pressure was increased at a steady rate of 30 kPA/s until the subject indicated that the pressure was “first perceived as painful”.

Participants then underwent an assessment of mechanical temporal summation using weighted pinprick stimulators, as in several previous studies [23]. The lowest-force stimulator that produced a sensation of pain (128 or 256 mN for most subjects) was used to apply a train of 10 stimuli to the skin on the dorsum of the hand at the rate of 1 per second. Participants rated the painfulness of the first, fifth, and tenth stimulus, and also rated any ongoing pain after-sensations 15 s following the final stimulus.

Response to deep pressure pain was ascertained via cuff pressure algometry (CPA). Tonic, deep-tissue, mechanical stimulation was applied using a Hokanson rapid cuff inflator; a standard blood pressure cuff was wrapped comfortably around the gastrocnemius muscle, and pressure was increased at approximately 5 mmHg/s. Participants indicated when the pressure was “first perceived as painful”.

Finally, responses to noxious cold were evaluated using a repeated cold pressor task (CPT), involving immersion of the right hand in 4 °C circulating water. Participants underwent 3 CPTs, with the first 2 consisting of immersions of the right hand for 30 s, with 2 min between immersions. The 3^rd^ and final CPT involved an immersion of the right hand lasting until a participant reached pain tolerance (or a 3 min maximum). Participants rated the maximum intensity of the cold pain on a 0–100 scale (“no pain” to “most intense pain imaginable”) at the conclusion of each CPT.

During the first 2 cold pressor tasks, we also assessed conditioned pain modulation (CPM), a non-invasive test of endogenous pain-inhibitory systems using a heterotopic noxious conditioning stimulation paradigm [24]. During the CPT, PPTh was assessed on the contralateral trapezius. CPM was quantified as percent change in PPTh during the CPT relative to baseline PPT. Cold pain intensity ratings (0–100) were also obtained at 30 s intervals following each CPT in order to quantify painful after-sensations.

### Shuttle Walking Test (SWT)

The SWT is a physical function task assessing how rapidly the patient can walk for 25 ft from a starting point and back. Patients were timed with a stopwatch and two runs were averaged. The SWT has shown good validity and reliability as an objective outcome measure of physical functioning [25].

### Data analysis

We compared OA patients to controls using independent group t-tests. Within the OA group, associations between QST responses and clinical pain outcomes were assessed using Pearson correlations. Repeated measures Analysis of Variance (ANOVA) was utilized to evaluate changes in pain outcomes. Associations between QST responses and changes in pain were assessed using Pearson correlations, with follow-up regression analysis in which baseline pain and demographic variables were controlled. Data were analyzed using SPSS 21 (IBM software, Chicago). Subjects with OA had to have completed at least 50 % of the study (i.e., at least 2 weeks of drug treatment) in order to be included in the statistical analysis.

## Results

**Fig. 1:**
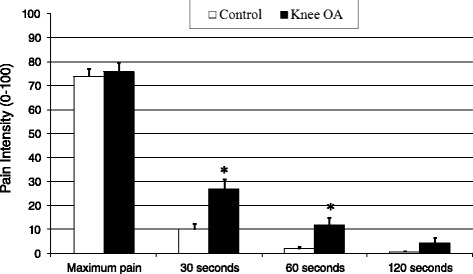
Cold pain ratings during and after cold pressor testing (data presented as means ± SEM). * Groups differ significantly at *p* < .05

**Fig. 2:**
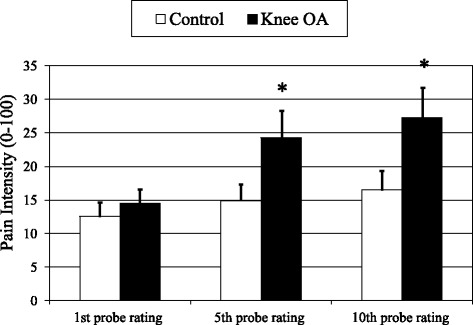
Pain ratings (0–100) for repetitive punctuate mechanical stimuli (data presented as means ± SEM). * Groups differ significantly at *p* < .05

**Fig. 3:**
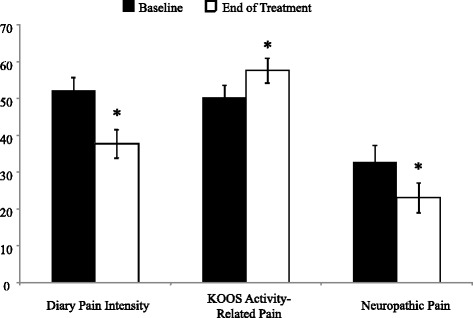
Pre-treatment and end-of-treatment scores on clinical outcome measures (data presented as means ± SEM). * Post-treatment differs from pre-treatment at *p* < .05

The groups were well-matched for demographic characteristics such as gender (knee OA group = 64 % women, control group = 67 % women) and age (patient age = 57.9 ± 10.7, control age = 59.8 ± 8.9). The controls were pain-free, while knee OA patients reported, on average, mild pain during the QST session and moderate levels of daily knee pain on the dairy and the KOOS (see Table 1 and Fig. 3). Consistent with prior results, the knee OA group showed significantly lower pain thresholds at anatomic locations near disease-affected sites (i.e., PPTh on the knee), and unaffected sites (i.e., PPTh on the trapezius) (*p*’s < .05). Tolerance for the cold pressor test was substantially lower among knee OA patients, and ratings of cold pain after-sensations were elevated (See Fig. 1) compared to controls (*p*’s < .05). In addition, CPM was lower in knee OA patients (See Table 1), while temporal summation of mechanical pain was enhanced (See Fig. 2) (*p*’s < .05).

**Table 1:** Comparison of psychophysical responses among knee OA patients (*n* = 35) and pain-free controls (*n* = 39)

	Knee OA (*n* = 35)	Controls (*n* = 39)
Variables of Interest Assessed During the QST Session		
Current Clinical Pain (0–100)	34.3 ± 26.2**	0 ± 0
Anxiety during QST (0–100)	23.1 ± 20.7 **	5.5 ± 7.1
Mechanical and Cold Pressor Responses		
PPTh (KPa)- Trapezius	275.1 ± 142.3*	346.4 ± 130.0
PPTh (KPa)- Thumb	227.5 ± 91.0**	339.9 ± 94.6
PPTh (KPa)- Knee	315.9 ± 158.0**	510.2 ± 188.6
Cuff Pain Threshold (mmHg)	129.3 ± 51.4*	168.6 ± 76.1
Cold Pain Tolerance (sec)	29.7 ± 30.8**	72.3 ± 60.5
Maximum Cold Pain (0–100)	75.9 ± 21.7	73.8 ± 22.2
CPM Index	118.7 ± 36.8*	142.5 ± 28.6
Other Mechanical and Cold Pressor Data Appear in Figs. 1 and 2		

*QST* quantitative sensory testing, *PPTh* pressure pain threshold, *KPa* Kilopascales, *CPM* conditioned pain modulation

*p < 0.05 for the group comparison

**p < 0.01 for the group comparison

We next examined, among patients, baseline relationships between QST responses and clinical pain measures. As expected, we observed moderate inter-correlations among these clinical pain measures: average diary pain intensity was correlated at *r* = −.61 with KOOS pain (higher scores on the KOOS indicate less pain, which is why these measures are negatively correlated), and at *r* = .65 with neuropathic pain symptoms, while KOOS pain and neuropathic pain symptoms were correlated at *r* = −.56 (all *p*’s < .001). Each pain scale was significantly correlated with SWT (i.e., for each measure, more severe pain is associated with longer walk times) at *p* < .05, with a mean correlation of *r* = .41. Overall, there were many significant relationships between QST responses and clinical measures (see Table 2). For example, cuff pain threshold on the gastrocnemius was significantly correlated (*p*’s < .01) with each measure of pain or function, with correlation coefficients of around .5, indicating roughly 25 % shared variance between mechanical pain sensitivity and the severity of OA pain and physical dysfunction.

**Table 2:** Correlations between QST measures and clinical pain at baseline among knee OA patients (*n* = 35)

QST Variables	Mean Daily Pain Intensity	Activity-Related KOOS Pain	Neuropathic Pain Symptoms	Shuttle Walk Time
PPTh- Trapezius	-.40*	.37*	-.43**	-.39*
PPTh- Thumb	-.33*	.37*	-.30	-.32*
PPTh - Knee	-.33*	.30	-.45**	-.42**
Cuff Pain Threshold	-.49**	.53**	-.49**	-.56**
Cold Pain Tolerance	-.21	.14	-.22	-.32*
Maximum Cold Pain	.33*	-.13	.24	.16
CPM Index	.10	-.10	.28	.08
Temporal Summation	.27	-.19	.31*	.24
Cold Pain After-Sensations	.34*	-.33*	.39*	.25

*QST* quantitative sensory testing, *PPTh* pressure pain threshold, *KPa* Kilopascales, *CPM* conditioned pain modulation

*p < 0.05 for the group comparison

**p < 0.01 for the group comparison

### Treatment-Related Changes in Pain Responses

Electronic diary monitoring was used to track the frequency of gel application; on average the cohort used the gel 3.4 times ± .6 per day. Repeated measures Analysis of Variance (ANOVA) indicated that each of the three clinical pain measures declined significantly over time: daily diary pain intensity F (1, 29) = 25.7, *p* < .001, KOOS activity-related pain F (1, 29) = 6.1, *p* < .05 and neuropathic symptoms F (1, 29) = 4.9, *p* < .05 (See Fig. 3).

QST responses were highly stable over time, with no changes, on average, from baseline to the final week of treatment (all *p*’s > .2). In order to reduce the number of analyses, we converted QST values to standard scores [26] and averaged the 3 values for PPTh (assessed at the thumb, trapezius, and knee) and the 3 cold pain variables (maximum cold pain rating, cold pain after-sensations, and cold pain tolerance, which was reverse-scored). Individual standard scores were used for cuff pain threshold, temporal summation, and CPM. Of the QST variables, only CPM was significantly associated with treatment response. CPM was negatively correlated with change in diary pain intensity (*r* = −.38, *p* < .05), positively correlated with change in KOOS (*r* = .45, *p* < .01) and negatively correlated with change in neuropathic pain symptoms (*r* = −.40, *p* < .05). In each case, higher baseline CPM scores were associated with more pain reduction at the end of treatment. We followed up these significant correlational results with linear regression analyses in which baseline CPM was examined as a predictor of end-of-treatment pain after controlling for baseline pain scores as well as age and gender. In the case of KOOS activity-related pain and electronic diary-reported daily pain intensity, baseline CPM was a significant unique predictor of end-of-treatment outcomes (See Table 3).

**Table 3:** Linear regression models predicting end-of-treatment Diary Pain Intensity and KOOS Activity-Related Pain

DV = Average Daily Pain Intensity				
Variable		Step R^2^	β	*p*
Step 1	Baseline Pain Intensity	.48** (for step)	.77**	.001
	Age		-.03	
	Sex		.21	
Step 2	Baseline CPM	.09* (for step)	-.30*	.03
DV = Koos Pain				
	Variable	Step R^2^	β	*p*
Step 1	Baseline KOOS Pain	.30** (for step)	.47**	.02
	Age		-.05	
	Sex		.16	
Step 2	Baseline CPM	.13* (for step)	.37*	.03

*CPM* conditioned pain modulation, *DV* dependent variable, *KOOS* knee injury and osteoarthritis outcomes score

*p < 0.05

**p < 0.01

## Discussion

The pathophysiology of OA pain is complex, with significant inter-individual variability in symptomatology that is unaccounted for by variation in radiographically-assessed joint damage or other traditional OA pain “mechanisms.” What is clear, however, is that central nervous system processes characterized by sensitization and maladaptive changes in endogenous pain modulation play a significant role in shaping clinical pain symptoms (2;3;8). A recent meta-analysis included 23 case-control studies (comparing OA patients to controls) with over 1,000 total subjects [27]. The authors concluded that pain thresholds were reduced in OA patients compared to controls in affected anatomic sites (e.g., the knee), distal sites adjacent to painful body locations, and also at remote sites. The authors attributed these findings to “spreading sensitization” or “central sensitization”, and suggested that QST should be used to phenotype OA patients into subgroups which might differ in treatment response.

The present study extends these findings by examining the capacity of dynamic QST phenotypes to predict responsiveness to a commonly-applied treatment. Compared to controls, the knee OA group demonstrated mechanical hyperalgesia in the form of reduced pain thresholds at affected sites such as the knee, distal sites such as leg muscles, and unaffected sites such as the trapezius. Indices of endogenous pain-modulatory processes also suggested potential decrements in pain inhibition. The prolongation of painful after-sensations, amplified temporal summation, and reduced CPM may indicate a relative predominance of facilitation over inhibition in central pain processing in knee OA patients [10]. Whether such group differences in pain-modulatory processes represent pre-existing risk factors that contribute to the development of chronic OA pain, or whether they are the consequence of chronic pain, is currently unknown. However, multiple prospective studies have now reported that successful joint replacement surgery is associated with “normalization” (e.g., improved CPM) of QST findings post-operatively [11, 12].

Measures of pain threshold and pain modulation were cross-sectionally related to individual variation in patient-reported OA pain. These associations indicated that the most pain-sensitive patients (i.e., those with the lowest pain thresholds), the patients with the greatest pain-facilitatory processes (e.g., the most temporal summation), and the least effective pain inhibition (i.e., the lowest CPM scores) tended to report the most severe pain. Other researchers have noted similar findings in OA patients [10, 28]. Such results highlight the possibility that QST phenotyping will prove useful in subgrouping patients across diagnostic categories. Interestingly, neuropathic pain symptoms were quite prevalent in this sample of knee OA patients, and these symptoms were responsive to treatment with a peripheral-acting, topical NSAID. The magnitude of these baseline neuropathic symptoms did not predict treatment response, though there were significant cross-sectional correlations with some QST measures of pain sensitivity, including a positive association with temporal summation of mechanical pain.

Prior studies have hinted at the neurochemical systems that may be involved in mediating CPM and temporal summation [10, 29, 30]. CPM, a sensitive measure of deficits in pain modulation in fibromyalgia and related persistent pain disorders, appears to depend on systems that interact with serotonergic and noradrenergic descending inhibitory pathways [31]. Temporal summation, an analog of central sensitization, represents an important pathophysiological process that contributes to the development and maintenance of pain states in a number of clinical contexts. While the temporal summation of pain involves processes at the spinal level, recent functional neuroimaging studies have highlighted the clear contribution of supraspinal processes as well [32, 33]. Collectively, the modulation of temporal summation appears to involve the activity of descending pain-inhibitory systems, which are known to play crucial roles in pain processing. In prior studies, medications such as NMDA antagonists, GABA agonists, and opioids have all been shown to reduce temporal summation of pain [34, 35]. Thus, the amplification of pain sensitivity in this sample of OA patients may reflect the activity of multiple neuroanatomical and neurochemical systems.

CPM may have an important prognostic role in forecasting treatment outcomes, and a recent study suggested that pre-treatment CPM scores predicted the analgesic efficacy of duloxetine [36]. Interestingly, diabetic neuropathy patients expressing lower baseline CPM scores seem to respond better to duloxetine. The authors noted that CPM reflects the functioning of descending pain-inhibitory pathways which utilize serotonin and norepinephrine. Therefore, a SNRI should be most effective in patients whose serotonergic and noradrenergic pain control systems function poorly at baseline. A more recent placebo-controlled trial of tapentadol, also in diabetic neuropathy patients, found that patients randomized to tapentadol subsequently improved their CPM, and this increase in CPM corresponded to the degree and temporal course of patients’ reduction in their neuropathic pain [37]. In our study, the knee OA patients demonstrating the largest magnitude of CPM at baseline also reported the most pain reduction during topical diclofenac treatment. This dissociation (i.e., those with poor CPM respond relatively better to an SNRI while those with better CPM respond relatively better to a peripherally-acting NSAID) highlights the potential utility of this sort of phenotyping in the process of treatment selection, as well as in understanding the mechanisms of treatment response and physiological characteristics of responders and non-responders to treatment.

A number of limitations should be considered when interpreting these findings. First, the sample size was relatively small, which precludes us from evaluating the unique predictive capacity of a broad set of factors. Second, the present study design did not include a control group, which prevents us from drawing definitive conclusions regarding the specific effects of NSAIDS on pain in this sample. Third, the duration of the study was rather short, limiting our analyses to the prediction of acute treatment responses. In other settings, CPM has been observed to predict longer-term outcomes such as the development of chronic pain 6 months after a surgery [31], and future studies of CPM’s predictive capacity may benefit from longer follow-up periods. Finally, while we did perform a multimodal QST assessment, we measured CPM in only one manner, and it is possible that differing patterns of findings might have obtained when using other methods.

## Conclusions

Despite these limitations, this study supports the clinical relevance of widespread hyperalgesia and maladaptive pain modulation in OA patients. Moreover, these findings are among the first to suggest that pre-treatment variation in CPM may be a valuable predictor of responses to analgesic treatments, regardless of the presence of neuropathic pain symptoms. Future treatment studies in OA may strongly benefit from baseline patient phenotyping using a QST protocol that includes CPM assessment. Overall, the findings of this study add to a small but promising body of literature supporting the capacity of psychophysical tests of pain modulation to provide useful, prospective, treatment-relevant information.

## Abbreviations

ANOVA, analysis of variance; CPA, cuff pressure algometry; CPM, conditioned pain modulation; CPT, cold pressor task; KOOS, knee injury and osteoarthritis outcomes score; KPa, Kilopascales; NSAID, nonsteroidal anti-inflammatory drug; OA, osteoarthritis; PPTh, pressure pain threshold; QST, quantitative sensory testing; SNRI, serotonin-norephinephrine reuptake inhibitor; SWT, shuttle walking test
